# Supplementary material for: Content-rich biological network constructed by mining PubMed abstracts
Source: BMC Bioinformatics. 2004 Oct 8;5:147. doi: 10.1186/1471-2105-5-147 (PMC528731; doi:10.1186/1471-2105-5-147)
Supplement: Additional File 2 — The original results of the above study (non-essential files are deleted to keep the file size under the limit set by BMC bioinformatics). [file 1471-2105-5-147-S2.bz2 › chilibotAdditionalFile2/dip10/704ID10734142E18041/html/MUTL_MUTS.html]

 


 **MUTL** and **MUTS** 
  
Found 186 abstracts in PubMed, retrieved 10.  
 

 What does Google say? 
 PDF only 
| .edu only 

---

**Interactive relationship** (e.g. stimulation, inhibition, etc)

**Neutral relationship**- Human mismatch repair protein  **MutL**  homologue 1 MLH1 interacts with Escherichia coli  **MutL**  and  **MutS**  in vivo and in vitro a simple genetic system to assay MLH1 function.  Ref: 12513688 Biochem J, 2003
- Both pathways require the formation of a  **MutS**  homolog complex which interacts with a second heterodimer, composed of two  **MutL**  homologs.  Ref: 12836196 Med Sci Paris , 2003

**Non-interactive relationship** (e.g. studied together, co-existance, homology, etc.)

- Protein components responsible for these reactions are highly conserved through evolution, and homologs of bacterial  **MutS**  and  **MutL** , which are key players in the initiation steps of both the strand specific mismatch correction and MMR dependent apoptotic signaling, have been identified in human cells.  Ref: 12700127 Front Biosci, 2003
- Finally, every mutation that abolishes mismatch repair is deficient in blocking homeologous recombination, suggesting that mismatch repair and prevention of homeologous recombination use the same  **MutS**   **MutL**  complexes for signaling in E. coli.  Ref: 12606120 DNA Repair Amst , 2003
- As compared to native  **MutS** , the ability of MutS800 to promote  **MutL**  dependent activation of MutH is substantially reduced.  Ref: 12829697 J Biol Chem< MedlineTA>J Biol Chem, 2003
- In eukaryotes, homologs of the Escherichia coli  **MutS**  and  **MutL**  proteins are crucial for both meiotic recombination and post replicative DNA mismatch repair.  Ref: 12836196 Med Sci Paris , 2003
- Exonuclease 1 Exo1 is a 5 3 exonuclease that interacts with  **MutS**  and  **MutL**  homologs and has been implicated in the excision step of DNA mismatch repair.  Ref: 12629043 Genes Dev, 2003
- These effects parallel the  **MutL**  and  **MutS**  binding and ATP binding hydrolysis activities of the mutated proteins.  Ref: 12513688 Biochem J, 2003
- The  **MutS**  homologous MSH and  **MutL**  homologous MLH proteins are highly conserved among all prokaryotes and eukaryotes.  Ref: 12582547 Theor Appl Genet< MedlineTA>Theor Appl Genet, 2003
- We have used the recently determined crystal structures of Escherichia coli E. coli  **MutS** ,  **MutL**  and MutH to guide construction of 47 amino acid substitutions in these proteins and analyzed their behavior in mismatch repair and recombination in vitro and in vivo.  Ref: 12606120 DNA Repair Amst , 2003
- In eukaryotic cells, DNA mismatch repair is initiated by a conserved family of  **MutS**  Msh and  **MutL**  Mlh homolog proteins.  Ref: 12529393 Mol Cell Biol, 2003
- The mutator phenotype was due to the ability of hMLH1 protein to interact with bacterial  **MutL**  and  **MutS**  proteins, thereby interfering with the formation of complexes between MMR proteins and mismatched DNA.  Ref: 12513688 Biochem J, 2003
- Strand specificity and repair efficiency are largely independent of the GATC methylation state of the DNA and presence of the products of mismatch repair genes mutH,  **mutL** , and  **mutS** .  Ref: 12692132 J Biol Chem< MedlineTA>J Biol Chem, 2003
- The human mismatch repair hMMR system consists of members of three super families, namely MutH,  **MutL**  and  **MutS** .  Ref: 12578652 Zhongguo Shi Yan Xue Ye Xue Za Zhi< MedlineTA>Zhongguo Shi Yan Xue Ye Xue Za Zhi, 2003
- In vitro and in vivo studies of  **MutS** ,  **MutL**  and MutH mutants correlation of mismatch repair and DNA recombination.  Ref: 12606120 DNA Repair Amst , 2003
